# Increased lateral femoral condyle ratio measured by MRI is associated with higher risk of solitary meniscus injury

**DOI:** 10.3389/fbioe.2024.1286967

**Published:** 2024-02-06

**Authors:** Lei Yang, Shuxing Xing

**Affiliations:** ^1^ Department of North Sichuan Medical College, Nanchong, Sichuan, China; ^2^ Department of Orthopedics, Chengdu Fifth People’s Hospital, Chengdu, Sichuan, China

**Keywords:** Meniscus injury, lateral femoral condyle ration, anatomical morphology of the knee, tibial plateau slope, MRI

## Abstract

**Background:** Past studies found that an increased lateral femoral condyle ratio is associated with anterior cruciate ligament injuries, but it is not clear if there is a link between MRI-measured lateral femoral condyle ratios and meniscal injuries. MRI provides a more accurate selection of measurement planes. Compared to X-rays, it further reduces data errors due to non-standard positions.

**Objective:** To study the relationship between knee bone morphology and Solitary meniscal injuries by MRI.

**Methods:** A total of 175 patients were included in this retrospective case-control study, including 54 cases of pure medial meniscus injury, 44 cases of pure lateral meniscus injury as the experimental group, and 77 control subjects. MRI images were used to measure the femoral notch width, femoral condylar width, femoral notch width index, lateral femoral condylar ratio (LFCR), posterior tibial slope, medial tibial plateau depth, and meniscus slope. In addition, carefully check for the presence of specific signs such as bone contusions and meniscal extrusions. Comparing the anatomical differences in multiple bone morphologies between the two groups, a stepwise forward multifactorial logistic analysis was used to identify the risk factors for Solitary meniscal injuries. Finally, ROC curves were used to determine the critical values and best predictors of risk factors.

**Results:** MTS, LTS, and LFCR ended up as independent risk factors for meniscus injury. Among all risk factors, LFCR had the largest AUC of 0.781 (0.714–0.848) with a threshold of 72.75%. When combined with MTS (>3.63°), diagnostic performance improved with an AUC of 0.833 (0.774–0.892).

**Conclusion:** Steep medial tibial plateau slope, steep lateral tibial plateau slope angle, and deep posterior lateral femoral condyles on MRI are independent risk factors for meniscal injuries. In patients with knee discomfort with the above imaging findings (X-ray, MRI), we should suspect and carefully evaluate the occurrence of meniscal injuries. It not only provides a theoretical basis to understand the mechanism of meniscus injury but also provides theoretical guidance for the prevention of meniscus injury and the development of intervention measures. Level of evidence III.

## Introduction

Currently, meniscus injuries are one of the most common sports injuries of the knee joint, resulting in poor late healing due to the specificity of its blood supply (Adams et al., 2021). The injury results in biomechanical disruption of the knee and worse long-term clinical outcomes (Zhou, 2018; Li et al., 2020a; Bradley et al., 2023). Therefore, more and more scholars have begun to pay more attention to the prevention and treatment of meniscal injuries and to understand the risk factors for meniscal injuries (Snoeker et al., 2013; Wu et al., 2022). Early assessment and identification are necessary to reduce the incidence of injury and the cartilage wear and early osteoarthritis of the knee caused by long-term injury.

With a deeper understanding of the role of knee joint bone morphology in the biomechanics of knee motion, the study of the correlation between tibiofemoral joint bone morphology and knee athletic injuries has received extensive attention. Recently, some knee anatomical morphologies such as posterior tibial slope (PTS), medial tibial plateau depth, and femoral condyle morphology have been recognized as potential risk factors for meniscal injuries and anterior cruciate ligament (ACL) injuries or graft failure (Li et al., 2021a; Jiang et al., 2022; Kim et al., 2022; Wang et al., 2022; Kodama et al., 2023). Increased tibial slope may result in increased anterior knee displacement and altered meniscal stress distribution (Kodama et al., 2023). The shallow depth of the medial tibial plateau results in less resistance to the femur sliding backward during flexion, thereby predisposing to increased relative motion of the tibiofemoral joint (Okazaki et al., 2021). The intercondylar fossa of the femur, as a conduit for the ACL, and the lateral femoral condyle (LFC), as a stopping point of the ACL, have been found in more studies to play important roles in ACL injuries (Shen et al., 2018; Li et al., 2020b). Asymmetry of the medial and lateral compartments of the knee increases knee rotational activity, and past studies have reported that posterior femoral condylar offset affects changes in knee kinematic range and carrier mechanics. Pfeiffer reported that the mechanism of the influence of lateral femoral condyle shape on knee rotation stability was an increase in the depth of the lateral femoral posterior condyle, and that the posterior condylar depth of the lateral femoral condyle was measured quantitatively (quantified as the lateral femoral condyle ratio) on a standardized lateral radiograph (Malviya et al., 2009; Pfeiffer et al., 2018; Hodel et al., 2019). In recent years, studies have investigated the relationship between x-ray measured lateral femoral condyle ratio (LFCR) and meniscal injuries, and found that deep posterior lateral femoral condyles (i.e., a larger lateral femoral condyle ratio) are an important risk factor for ramp lesions (Kim et al., 2021). However, whether the LFCR measured by MRI is a potential risk factor for meniscus is not clear. MRI provides a more accurate selection of measurement planes. Compared to X-rays, it further reduces data errors due to non-standard positions.

In addition, ACL injuries are often accompanied by meniscal injuries, so previous researchers often chose patients with ACL combined with meniscal injuries as the study subjects to explore the relationship between knee joint bone morphology and meniscal injuries (Kodama et al., 2023). The ACL has a function in the knee joint of limiting forward over shift and internal rotation of the tibia, and ACL injury can lead to instability in the knee joint (Shen et al., 2018). Therefore, the relationship between knee bone morphology and meniscus injury is not reflected in such conditions. Therefore, patients with solitary meniscus injuries were selected in this study to determine whether the ratio of the lateral femoral condyle and other knee imaging parameters in the MRI condition are associated with meniscus injuries. We hypothesized that solitary meniscal injuries are associated with knee bone morphology including LFCR and tibial slope. This study can help us understand the potential risk factors for meniscal injuries and help in the early assessment and implementation of prevention strategies.

## Materials and methods

This is a retrospective case-control study that included patients who had visited our hospital for knee pain from January 2022 to June 2023, all of whom underwent a 3.0 T (“T” stands for Tesla, a unit of magnetic field strength) MRI of the knee. All patients were diagnosed by MRI imaging and the diagnosis was confirmed by an orthopedically experienced joint surgeon. Inclusion criteria included 1) high signal (at least grade II injury) in the meniscus on MRI; 2) No manifestation of ACL or other peripheral ligament injury on MRI. Exclusion criteria included 1) age <18 years or >50 years; 2) the presence of periprosthetic ligament and bony structure injuries of the knee; 3) the presence of patients with gouty arthritis, rheumatoid arthritis, and osteoarthritis of the knee; 4) the presence of a history of knee surgery, history of violent traumatic injuries; and 5) the presence of substandard MRI imaging scans. The clinical study was conducted by the Declaration of Helsinki and the relevant ethical requirements of the Fifth People’s Hospital of Chengdu. All patients gave fully informed consent for imaging examinations and data collection.

### MRI measurement

All patients were scanned with 3.0 T MRI, with the patients in a supine position with the knee naturally straightened and the toes pointing upward, and the scans included axial, sagittal, and coronal positions, respectively. The scanning parameters were 1) axial: repetition time (TR) 2,577 ms, echo time (TE) 45 ms, field of view (FOV) 16 × 16 mm, thickness of slice 3–4 mm; 2) sagittal: repetition time (TR) 584 ms, echo time (TE) 20 ms, field of view (FOV) 16 × 16 mm, thickness of slice 3–4 mm; 3) coronal: repetition time (TR) 2,345 ms, echo time (TE) 45 ms, field of view (FOV) 16 × 16 mm, thickness of slice 3–4 mm.

Knee MRI data were collected from all included cases and DICOM images were opened and measured using Mimics 21.0 software. Meniscal injuries were independently judged by two experienced orthopedic surgeons and classified using the three-tiered staging system on the T2 signal mentioned by Fischer et al. (1991): I. Presence of a punctate confined high signal shadow; II. Presence of a linear high signal shadow without involvement of the articular surface; III. High-signal shadow involving at least one articular surface. Knee anatomical morphologies such as femoral notch width, femoral condylar width, femoral notch width index, tibial slope, medial tibial plateau depth, meniscus slope, and LFCR were measured by two experienced orthopedic surgeons(X.S.X., Y.L.) on MRI images. To assess the reliability of the measurements, 30 patients were randomly selected and the measurements were repeated 3 weeks later by a physician (Y.L). All cases were measured without knowledge of the measurement and the intragroup correlation coefficient (ICC) was calculated to evaluate the reliability of the measurement. The ICC values < 0.8 were considered poor, between 0.8 and 0.9 were considered good, and ICC values > 0.9 were considered excellent (Song et al., 2018).

**FIGURE 1:**
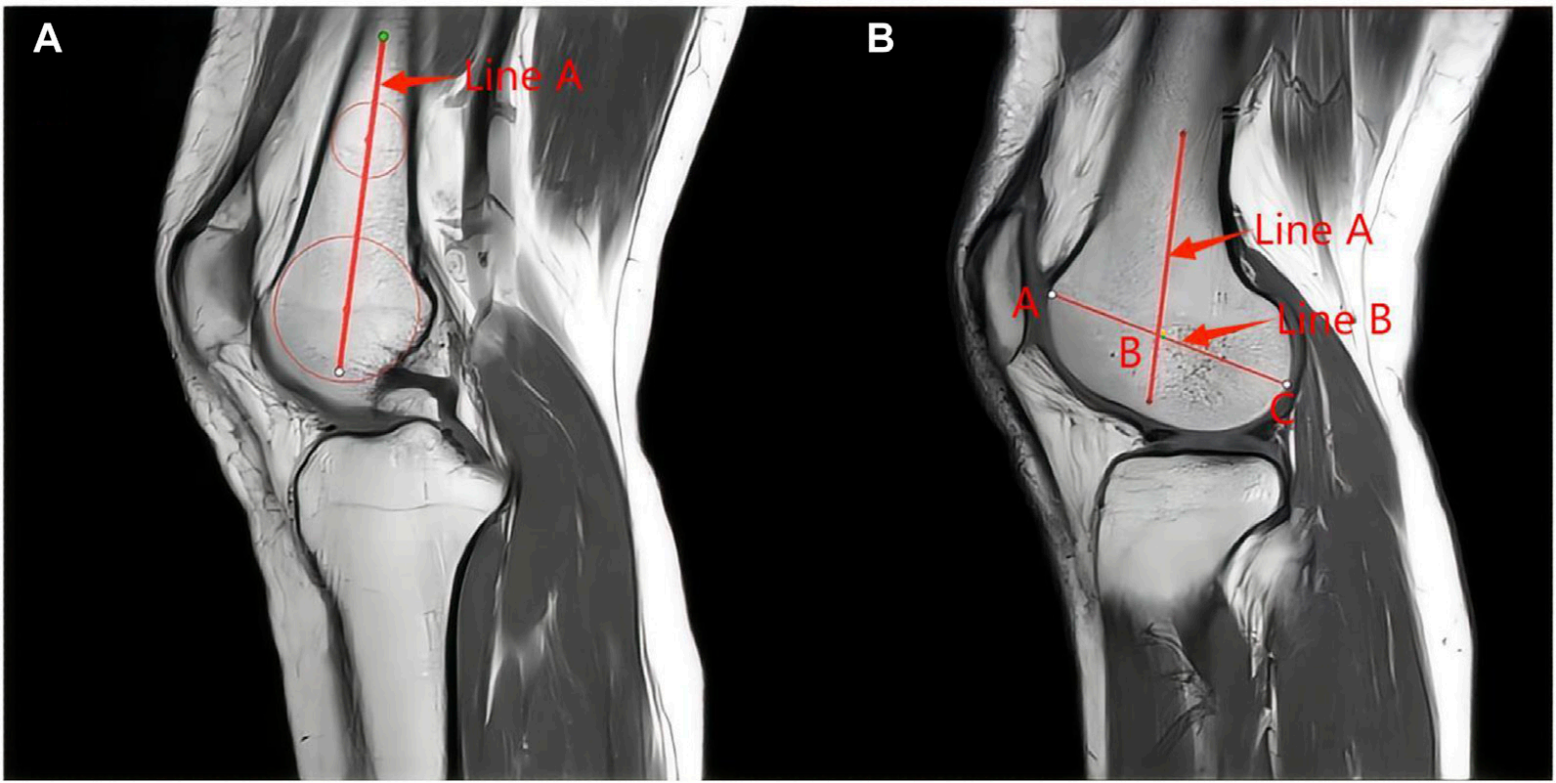
Measurement of LFCR: **(A)** In the sagittal T1 center of the knee MRI, 2 circles were drawn in the center of the femoral axis to determine the long axis of the distal femur. The more distant circle was placed at the nearest end of the tackle. The line passing through the center of the two circles was considered to be the long axis of the distal shaft of the femur (line A). **(B)** In the sagittal T1 MRI center of the lateral condyle of the femur, the long axis of the distal femur was replicated (line A). The axis of the femoral condyle was then determined by drawing a line (line B) between the last point of the lateral condyle and the most anterior point. The distance from the intersection of the two lines (point B) to the last point of the condyle was divided by the total length of the condyle (BC/AC). This ratio was defined as the lateral femoral condyle ratio.

LFCR is referenced to the measurements of Kim et al. (2021), He and Li (2022) (Figure 1). First, a central sagittal plane containing the posterior cruciate ligament terminus, intercondylar spine, and anterior and posterior tibial cortical depressions on MRI of the knee was selected. To determine the anatomical axis of the distal femur, two circles are drawn on the distal femur, with the most distal end placed near the femoral condyles, passing through the centers of the two circles is the anatomical axis of the distal femur. In the central sagittal plane of the T1MRI of the lateral femoral condyle, the anatomical axis of the distal femur was replicated and a line was drawn through the lateral femoral condyle between the most anterior point and the last point for the axis of the lateral femoral condyle, and the distance from the intersection point of the two lines to the posterior condyle was calculated as the LFCR by dividing it by the total length.

**FIGURE 2:**
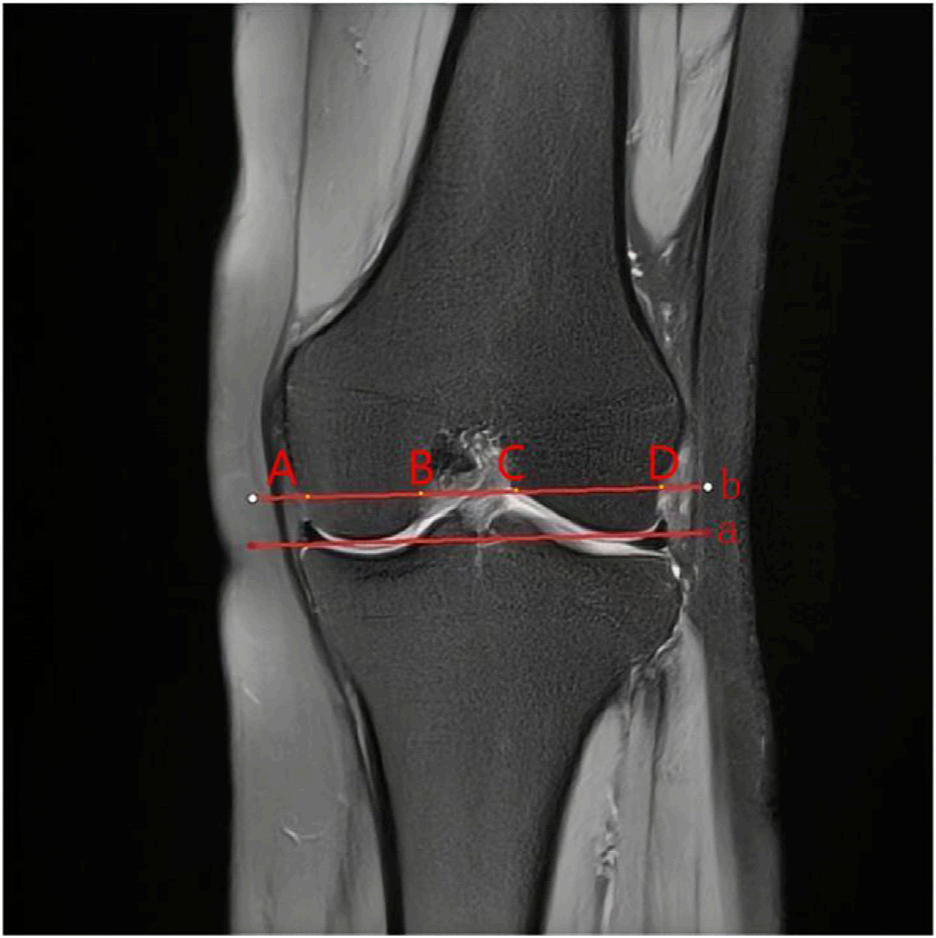
Measurement of femoral condylar parameters. “a” line was then drawn through the medial and lateral lowermost margins of the femoral condyles and a parallel line (b) was drawn at the level of the popliteal groove. **(A,D)** and **(B,C)** in Figure 1 are FCW and NW widths, NWI = NW/FCW.

**FIGURE 3:**
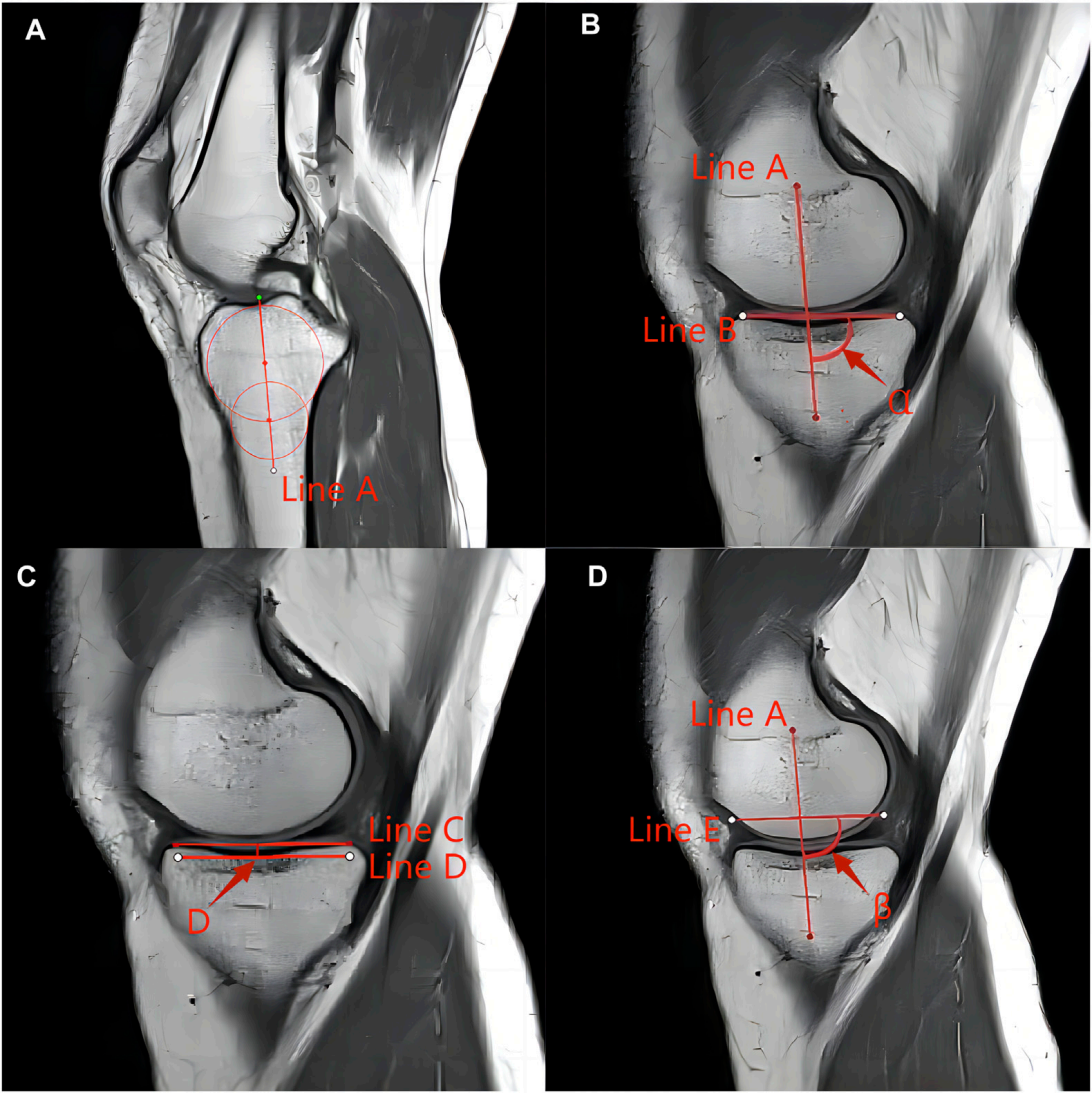
Measurement techniques of determining the posterior tibial slope in the MRI according to the method described by Hudek et al. **(A)** Determination of the proximal anatomical axis of the tibia. Localization of the central sagittal plane is performed in the sagittal position. The plane includes the posterior cruciate ligament termination point, the intercondylar eminence, and the anterior and posterior tibial cortical concave. Two circles are drawn on the proximal tibia, the proximal circle is tangent to the anterior and posterior tibial cortex and tibial plateau, and the distal circle is tangent to the anterior and posterior tibial cortex, and the center of the circle is on the proximal circle center. The center of the circle connecting the two circles is the anatomical axis of the proximal tibia (line A). **(B)** Measurement of the posterior slope angle of the tibial plateau: determine the middle sagittal plane of the medial tibial plateau (the midpoint from the intercondylar spine to the edge of the medial tibia), connect the anterior and posterior margins of the uppermost tibia with the cortex, and draw a tangent line to the medial plateau (line B). Duplicate the proximal tibial anatomical axis in this plane (line A), and measure the angle between the vertical line of the proximal tibial anatomical axis and the medial tibial plateau tangent as the medial tibial plateau posterior tilt angle (90°-α). Similarly, the posterior inclination of the lateral tibial plateau was measured in the same way. **(C)** Depth of the medial tibial plateau: in the middle sagittal plane of the medial tibial plateau, connecting the uppermost anterior and posterior margins of the tibial plateau with the cortex, draw a tangent line of the medial plateau (Line C), and then at the lowest point of the concave surface of the tibial plateau draw a tangent line parallel to this line (Line D), which represents the lowest boundary of the subchondral bone, and measure the perpendicular distance between the 2 lines as the depth of the medial tibial plateau (“D” in Figure 3c). **(D)** Measurement of posterior slope of the meniscus: in the sagittal plane in the middle of the medial tibial plateau mentioned above, connect the anterior and posterior corners of the meniscus (Line E), copy the anatomical axis of the proximal tibia in this plane, and measure the angle between the plumb line of the proximal tibial anatomical axis and the tangent line of the meniscus, i.e., the posterior inclination of the medial meniscus (90°-β). Similarly, the posterior inclination of the lateral meniscus was measured in the same way.

In this study, other knee bone morphology was measured in previous studies. Femoral notch width, femoral condylar width, and femoral notch width index were measured using the method described by Domzalski et al. (2010), Wang et al. (2022) (Figure 2). The tibial slope, medial tibial plateau depth, and meniscus slope were used by Hudek et al. (2009), Li et al. (2021b) (Figure 3). Bone contusion and meniscus extrusion were judged by Beel et al. (2022), Kim et al. (2022). Bone contusion is a contusion of the medial and lateral tibia and femur that appears as a bright edematous shadow on the MRI T2 image. Meniscus extrusion is when the edge of the meniscus exceeds the edge of the medial or lateral tibia, and in this study, bone contusion and meniscus extrusion were recorded positively in agreement with the side of the meniscus injury.

### Statistical analysis

Statistical analysis was performed using IBM SPSS 27.0 with a significance level of 0.05. For continuous quantitative data, normal distribution was tested using the Kolmogorov-Smirnov test, and if conforming to the normal distribution data were expressed using the mean ± standard deviation, and independent samples t-tests were used to test the differences in each anatomical feature between the injury group and the normal group. Those that did not fit the normal distribution were expressed as median and interquartile spacing and analyzed using the Wilcoxon rank sum test. For qualitative data, the relationship between meniscus injury and bone contusion, and meniscus extrusion was analyzed using the chi-square test. Anatomical measurements with significant differences were selected for stepwise forward multifactorial logistic regression analysis, and ultimately MTS, LTS, and LFCR were identified as independent risk factors for solitary meniscus injury. Risk factors for medial and lateral meniscus injury were also analyzed separately. Screening for combinations of anatomical features that are good predictors of meniscal injuries was based on SPSS binary logistic results Omnibus test, -2-fold log-likelihood. The receiver operating characteristic curve (ROC) and area under the curve (AUC) were calculated to compare the predictive efficacy of each risk factor, and the optimal cutoff value was determined at the maximum Jordon index.

## Results

Finally, 175 patients were included (98 in the injury group and 77 in the normal control group). The injury group included 47 males and 51 females, and the control group included 33 males and 44 females. The injury group had a mean age of 32.90 ± 9.72 and a mean BMI of 23.08 ± 2.27; the control group had a mean age of 29.16 ± 7.98 and a mean BMI of 22.83 ± 2.70. Screening part of the indicators (MTS, LTS, MTD, LFCR) in 30 randomized patients were re-measured, and the inter-observer/intra-observer ICC was calculated to be 0.83/0.85, 0.82/0.87, 0.84/0.85, 0.84/0.90, respectively, and the ICC of all measurements was >0.8, which proved that the data were reliable.

**FIGURE 4:**
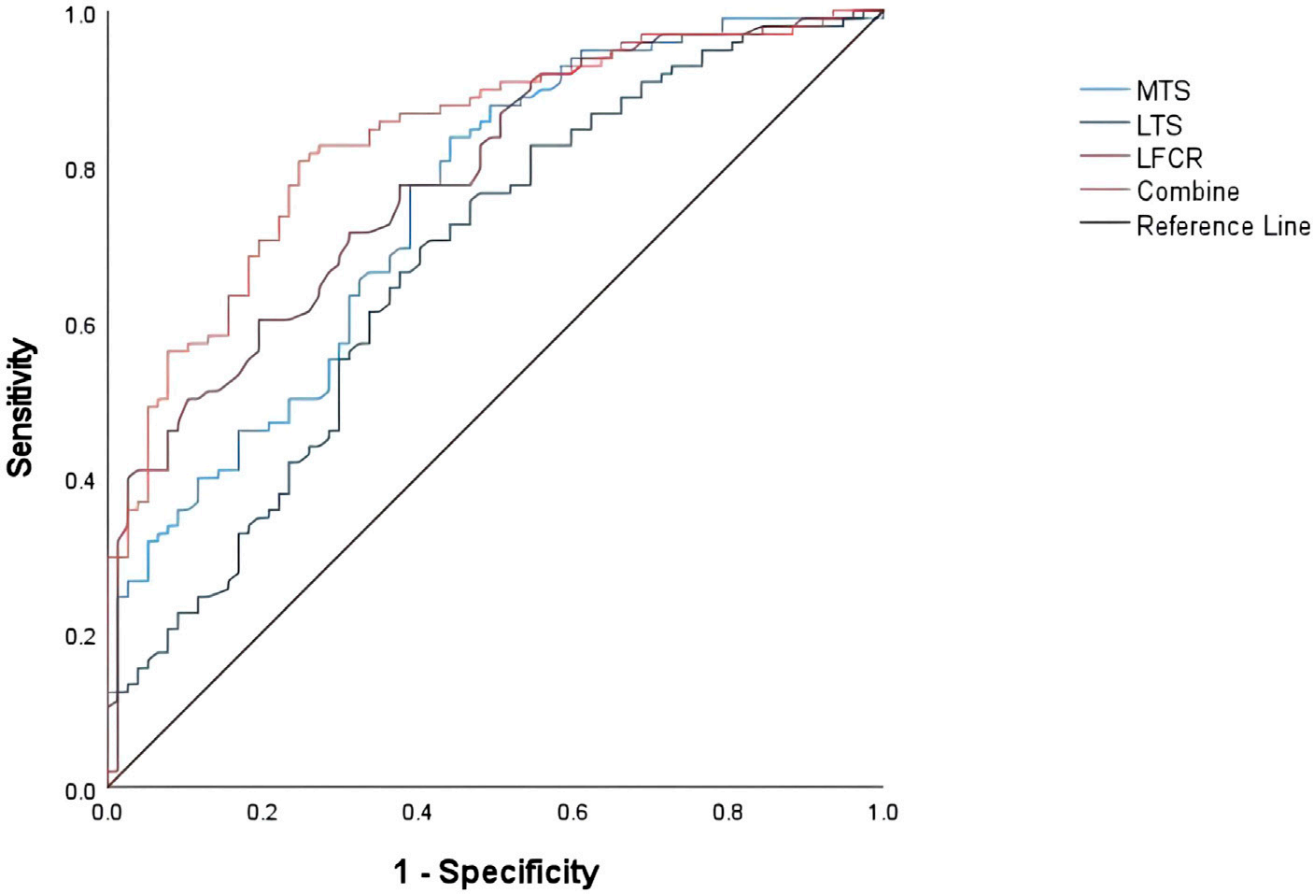
Receiver operating characteristic curves for MTS, LTS, LFCR, and MTS + LFCR. Reference line: AUC = 0.5. AUC is the area under the curve.

All anatomical features were analyzed by comparing the two groups, and the differences in MTS, LTS, LFCR, and meniscus extrusion were statistically different (*p* < 0.001). There was no statistically significant difference between the two groups for NW, FCW, NWI, MTD, and bone contusion. Demographic data and data on anatomical characteristics of the two groups are shown in Table 1. All indicators were analyzed in a binary logistic regression model, and three variables were found to be associated with meniscal injuries: the MTS [odds ratio (OR) 2.36, 95% CI (1.44–3.87)], the LTS [OR 1.76,95% CI (1.05–2.94)], and the LFCR [OR 1.25, 95% CI (1.07–1.46)], therefore three variables were all risk factors for meniscal injury. The results of the univariate and multivariate analysis of the anatomical characteristics of the knee joints of the two groups are shown in Table 2. Analysis of the ROC curve showed that the area under the curve (AUC) was 0.746 (0.674–0.819) for MTS, 0.675 (0.595–0.756) for LTS, 0.781 (0.714–0.848) for LFCR, and 0.833 (0.774–0.892) for MTS + LFCR. The cutoff values for MTS, LTS, and LFCR were 3.63° (sensitivity 76% and specificity 62%), 3.84° (sensitivity 66% and specificity 62%), and 72.75% (sensitivity 60% and specificity 81%), respectively (Figure 4). Multifactorial Logistic alone analyzed the independent risk factors for meniscal injuries in different locations. MTS and LFCR were risk factors for medial meniscus injuries with OR values of 4.99 (1.96–12.70) and 1.43 (1.13–1.80) respectively. LTS was a risk factor for lateral meniscus injuries with OR value of 2.43 (1.29–4.59). The results of risk factors for injuries at different locations of the meniscus are shown in Table 3.

**TABLE 1:** Demographic data and data on anatomical characteristics of the two groups.

Variable	Injury group (n = 98)	Control group (n = 77)	*p*-value
Age (years)	32.90 ± 9.72	29.16 ± 7.98	<0.001
BMI((kg/m2)	23.08 ± 2.27	22.83 ± 2.70	0.72
sex			
man	47	33	0.5
woman	51	44	
NW(mm)	19.77 ± 2.50	19.34 ± 2.57	0.27
FCW(mm)	69.05 ± 5.29	67.98 ± 5.93	0.21
NWI	0.29 ± 0.02	0.28 ± 0.03	0.56
MTS (°)	4.96 ± 1.40	3.67 ± 1.30	<0.001
LTS (°)	4.53 ± 1.42	3.65 ± 1.37	<0.001
MTD (mm)	1.48 ± 0.55	1.60 ± 0.59	0.17
LFCR(%)	73.60 ± 0.83	69.26 ± 4.19	<0.001
Bone bruise			
+	6	6	*p* = 0.68
−	91	71	
meniscus extrusion			
+	56	23	*p* < 0.001
−	41	54	

Note: NW, notch width; FCW, femoral condylar width; NWI, notch width index; LFCR, lateral femoral condylar ratio; MTS, medial posterior tibial slope; MTD, medial tibial plateau depth; LTS, lateral posterior tibial slope; MMS, medial posterior meniscus slope.

**TABLE 2:** The results of the univariate and multivariate analysis of the anatomical characteristics of the knee joints of the two groups.

Variable	Univariate analysis *p*-value	Multivariate analysis				
		β	Z.E	*p*-Value	OR	95%CI
Age (years)	0.001	0.08	0.06	0.142	1.09	0.97–1.22
MTS (°)	<.001	0.94	0.43	0.029	2.36	1.44–3.87
LTS (°)	<.001	0.56	0.26	0.031	1.76	1.05–2.94
LFCR(%)	<.001	0.22	0.08	0.005	1.25	1.07–1.46
Bone bruise	0.68	0.19	0.12	0.121	1.21	0.95–1.54
meniscus extrusion	<0.001	0.22	1.03	0.831	1.25	0.16–1.47
BMI((kg/m2)	0.72					
NW(mm)	0.27					
FCW(mm)	0.21					
NWI	0.56					
MTD (mm)	0.17					

**TABLE 3:** The results of risk factors for injuries at different locations of meniscus.

Medial meniscus injury			Lateral meniscus injury		
Variable	OR 95%CI	*p*-Value	Variable	OR 95%CI	*p*-Value
MTS	4.99 (1.96–12.70)	<0.001	LTS	2.43 (1.29–4.59)	0.006
LFCR	1.43 (1.13–1.80)	0.002			

## Discussion

The most important finding of this study was that the anatomical morphology of the knee was significantly associated with solitary meniscal injuries. Specifically, increased MTS, LTS, and LFCR were associated with solitary meniscal injuries compared with healthy controls, and MTS and LFCR were medial meniscus risk factors; LTS was a lateral meniscus risk factor. The ability to predict solely meniscal injuries was highest with LFCR (LFCR >72.75%) with an AUC of 0.781 (0.714–0.848), and the combination of LFCR + MTS (LFCR >72.75% and MTS >3.63) improved the predictive diagnostic performance for meniscal injuries with an AUC of 0.833 (0.774–0.892). Understanding These parameters can help clinicians more effectively identify patients who are at risk for meniscal injuries.

Previous studies have used different measurement tools and measurements to describe knee bone morphology parameters and further explored the association with anterior cruciate ligament or meniscus injuries (Kim et al., 2021; He and Li, 2022). X-rays have been used in previous studies to quantify bone morphology in the knee and are simple and easy to perform. X-rays require a standardized position for radiographs; otherwise, the selection and exclusion of study subjects are often affected by factors such as overlap and poor rotation, further affecting the conclusions (Voleti et al., 2014). Pfeiffer (Pfeiffer et al., 2019) quantified the lateral posterior femoral condylar depth (quantified as the lateral femoral condyle ratio) on standard lateral radiographs and demonstrated an association with ACL injuries or meniscus injuries as reported by investigators such as Li et al. (2021a) and Kim et al. (2021). Voleti et al. (2014) demonstrated that even though the trend of conclusions was consistent, X-rays underestimate LFCR measurements compared to MRI. MRI allows for more precise plane selection and accurate quantification of the depth of the posterior condyle of the lateral femoral condyle, assesses their influence on the risk of ACL injury, and reduces measurement imprecision and patient exclusion because of malrotation radiographs (Voleti et al., 2014; Hodel et al., 2019). Lateral femoral condyle morphology includes anteroposterior diameter, curvature, *etc.* Hodel et al. (2019) quantified the lateral femoral condyle morphology on MRI using the lateral femoral condyle index (LFCI), which is a flexion circle and an extension circle drawn anteroposteriorly and posteriorly to the lateral condyle of the femur, and the ratio of the radius of the two circles is considered to be the LFCI. It was found that LFCI is a risk factor for non-contact ACL injuries, but in Nowak et al. (2022) study such a relationship was not found to be valid, and the results were analyzed and found that this seems to be mainly due to methodological differences, as Li et al. (2020c) pointed out in his study that the determination of the two circles is highly influenced by the subjectivity of the person who takes the measurements, and it has a high degree of uncertainty and randomness. Therefore, it is particularly important to quantify the lateral femoral condyle morphology using an accurate and stable measurement method.

Many studies have shown that the lateral femoral condyle morphology exerts a significant influence on the rotational stability of the knee (Kujala et al., 1992; Fernandes et al., 2017). Pfeiffer et al. (2019) reported that the mechanism by which lateral femoral condyle morphology affects rotational stability of the knee may be that an increase in the lateral femoral posterior condyle results in a more elliptical and inequidistant lateral femoral condyle, which may alter tibiofemoral relative motion and result in altered knee kinematics and loading mechanics. The tibial stop of the anterolateral ligament is relatively fixed and the femoral stop is still controversial. It has been found that its stop is located on the convexity of the lateral epicondyle and is connected anteriorly to the lateral collateral ligament stop (Vincent et al., 2012; Kraeutler et al., 2018). Thus, deep posterior lateral femoral condyles may increase the length of the anterolateral complex, which may increase the rotational laxity of the knee. Kim et al. (2021) measured LFCR in a standard lateral X-ray of the knee and determined that a higher LFCR was a significant risk factor for Ramp lesions. Although there was a difference in the critical values obtained by MRI and X-ray (72.75% vs 71%), the correlation between the two was similar for meniscal injuries.

In knees with meniscal injuries, a steep tibial slope leads to increased forward translation of the tibia, resulting in abnormal stress distribution in the meniscus. As well, the meniscus serves as a secondary adjunct to knee stability and will be exposed to higher axial loads in an unstable knee (Beel et al., 2022; Kim et al., 2022; Cristiani et al., 2023; Kodama et al., 2023). Li et al. (2021b) reported that medial tibial slope and lateral tibial slope were risk factors for isolated medial meniscus injury (OR = 2.979) and lateral meniscus injury (OR = 2.391), respectively. Similarly, Okazaki et al. (2021) demonstrated that MTS (OR = 2.76) was a risk factor for medial meniscus posterior root tears. Wong et al. (2022) utilized the fact that increased LTS leads to worse clinical outcomes (IKDC) in patients after lateral meniscus repair, which seems to indirectly demonstrate that steep LTS is a risk factor for meniscal injury. Deng et al. (2021) used X-rays to measure tibial plateau slope and their results identified steep tibial plateau slope as a risk factor for meniscus injury. However, there have also been reports of no association between steep PTS and meniscal injuries (Beel et al., 2022). In this study, we found that the combination of two factors (LFCR + MTS) was more predictive of meniscal injury. This confirms the above findings that tibiofemoral joint bone morphology such as deep posterior lateral femoral condyles and steeper tibial slope increases alters knee biomechanics, resulting in greater stress on the meniscus and increased risk of injury.

Other knee bone morphology such as intercondylar fossa width and intercondylar fossa width index, although important anatomical parameters of the knee joint, were not found to be significantly associated with meniscal injuries in this study. This may be related to multiple factors: inconsistent selection of study participants, inconsistent measurement methods, *etc.* Patients with ACL accompanied by meniscus injuries are often selected as subjects in previous studies, which may generate false positive results (Li et al., 2021a). In this study, Solitary meniscus injury patients were selected for the study subjects and the incidence of bone contusion was only 6.8% Comparison of 82.1% incidence of bone contusion in the study by Beel et al. (2022) can further demonstrate the impact of ACL injury.

With this study, we made some meaningful findings. First, this study provides a theoretical basis for understanding the risk factors for meniscal injuries, assists clinicians in effectively identifying patients with risk factors, and contributes to the prevention and diagnosis of meniscal injuries, such as in this study MTS + LFCR may assist in identifying meniscal injuries. In addition, a comparative analysis of the present results with those under previous x-ray measurements revealed that the simplicity of the measurement tool may have led to some bias in the results, but the trend of correlation with meniscus injuries was approximately the same. Clinically, there is an increasing number of young patients with knee pain as their main symptom, but no obvious knee sports injury. These patients are characterized by long-term disease duration and untimely treatment, an MRI or arthroscopy of the knee joint reveals different degrees of damage to the patient’s articular cartilage, which affects the long-term prognosis of the patients (Le et al., 2023). The corresponding symptoms and signs combined with the variation of anatomical morphology on X-rays should attract our attention, and we should actively complete the MRI examination for early and specific diagnosis and further treatment. Finally, knee bone morphology as a non-modifiable factor guides the implementation of late rehabilitation programs for knee sports injuries such as ACL injuries, meniscus injuries, and other ligamentous muscle injuries by delaying the onset of weight-bearing and strengthening the quadriceps muscles in the expectation of a better prognosis.

There are some limitations in this study. First, all included cases in this study were diagnosed by MRI, and limited mucoid degeneration of the meniscus was difficult to differentiate from injury in grades I and II. Second, age was controlled below 50 years based on previous systematic reviews to limit the impact of osteoarthritis of the knee, but there was still an age mismatch between the two groups that may have influenced the results. Third, previous studies (Li et al., 2021b) have indicated that there are gender differences in knee bone morphology parameters, and further subgroup analyses by gender were not performed in this study, which may have had some impact on the results. Fourth, the knee MRI slice thickness in this study was 4 mm, which is less accurate for the required plane than a slice thickness of 2–3 mm (Amerinatanzi et al., 2017). Finally, this study still used a retrospective case-control study, and the correlation between the two still needs to be further verified, more prospective cohort studies and so on are needed at a later stage to confirm it. In recent years, some researchers have shown the relationship between multiple anatomical changes in the knee joint and meniscal injuries, but it has not been verified by biomechanical changes in the knee joint. This study also suffers from this shortcoming, therefore, we would like to use a finite element analysis model to explore the force distribution and force magnitude changes of the meniscus after different anatomical morphology changes in the subsequent work.

## Conclusion

Steep medial tibial plateau slope, steep lateral tibial plateau slope angle, and deep posterior lateral femoral condyles on MRI are independent risk factors for meniscal injuries. In patients with knee discomfort with the above imaging findings (X-ray, MRI), we should suspect and carefully evaluate the occurrence of meniscal injuries. It not only provides a theoretical basis to understand the mechanism of meniscus injury but also provides theoretical guidance for the prevention of meniscus injury and the development of intervention measures.

## Data Availability

The original contributions presented in the study are included in the article/Supplementary Material, further inquiries can be directed to the corresponding author.

## References

[B1] Adams B. G. Houston M. N. Cameron K. L. (2021). The epidemiology of meniscus injury. Sports Med. Arthrosc. Rev. 29 (3), e24–e33. 10.1097/JSA.0000000000000329 34398119

[B2] Amerinatanzi A. Summers R. K. Ahmadi K. Goel V. K. Hewett T. E. Nyman E. (2017). Automated measurement of patient-specific tibial slopes from mri. Bioengineering-Basel 4 (3), 69. 10.3390/bioengineering4030069 28952547 PMC5615315

[B3] Beel W. Mouton C. Tradati D. Nührenbörger C. Seil R. (2022). Ramp lesions are six times more likely to be observed in the presence of a posterior medial tibial bone bruise in acl-injured patients. Knee Surg. Sports Traumatol. Arthrosc. 30 (1), 184–191. 10.1007/s00167-021-06520-z 33661324

[B4] Bradley P. X. Thomas K. N. Kratzer A. L. Robinson A. C. Wittstein J. R. DeFrate L. E. (2023). The interplay of biomechanical and biological changes following meniscus injury. Curr. Rheumatol. Rep. 25 (2), 35–46. 10.1007/s11926-022-01093-3 36479669 PMC10267895

[B5] Cristiani R. van de Bunt F. Kvist J. Stålman A. (2023). High prevalence of meniscal ramp lesions in anterior cruciate ligament injuries. Knee Surg. Sports Traumatol. Arthrosc. 31 (1), 316–324. 10.1007/s00167-022-07135-8 36045182 PMC9859899

[B6] Deng X. Hu H. Song Q. Zhang Y. Liu W. Zhu L. (2021). The influence of the steep medial posterior tibial slope on medial meniscus tears in adolescent patients: a retrospective case-control study. BMC Musculoskelet. Disord. 22 (1), 901. 10.1186/s12891-021-04766-9 34696769 PMC8546944

[B7] Domzalski M. Grzelak P. Gabos P. (2010). Risk factors for anterior cruciate ligament injury in skeletally immature patients: analysis of intercondylar notch width using magnetic resonance imaging. Int. Orthop. 34 (5), 703–707. 10.1007/s00264-010-0987-7 20333378 PMC2903177

[B8] Fernandes M. S. Pereira R. Andrade R. Vasta S. Pereira H. Pinheiro J. P. (2017). Is the femoral lateral condyle's bone morphology the trochlea of the acl? Knee Surg. Sports Traumatol. Arthrosc. 25 (1), 207–214. 10.1007/s00167-016-4159-1 27161195

[B9] Fischer S. P. Fox J. M. Del P. W. Friedman M. J. Snyder S. J. Ferkel R. D. (1991). Accuracy of diagnoses from magnetic resonance imaging of the knee. A multi-center analysis of one thousand and fourteen patients. J. Bone. Jt. Surg. Am. 73 (1), 2–10. 10.2106/00004623-199173010-00002 1985991

[B10] He M. Li J. (2022). Increased lateral femoral condyle ratio measured by mri is associated with higher risk of noncontact anterior cruciate ligament injury. BMC Musculoskelet. Disord. 23 (1), 190. 10.1186/s12891-022-05134-x 35232401 PMC8886831

[B11] Hodel S. Kabelitz M. Tondelli T. Vlachopoulos L. Sutter R. Fucentese S. F. (2019). Introducing the lateral femoral condyle index as a risk factor for anterior cruciate ligament injury. Am. J. Sports. Med. 47 (10), 2420–2426. 10.1177/0363546519858612 31295005

[B12] Hudek R. Schmutz S. Regenfelder F. Fuchs B. Koch P. P. (2009). Novel measurement technique of the tibial slope on conventional mri. Clin. Orthop. Relat. Res. 467 (8), 2066–2072. 10.1007/s11999-009-0711-3 19190973 PMC2706341

[B13] Jiang J. Liu Z. Wang X. Xia Y. Wu M. (2022). Increased posterior tibial slope and meniscal slope could be risk factors for meniscal injuries: a systematic review. Arthroscopy 38 (7), 2331–2341. 10.1016/j.arthro.2022.01.013 35066109

[B14] Kim S. H. Park Y. B. Won Y. S. (2021). An increased lateral femoral condyle ratio is an important risk factor for a medial meniscus ramp lesion including red-red zone tear. Arthroscopy 37 (10), 3159–3165. 10.1016/j.arthro.2021.03.078 33892074

[B15] Kim S. H. Seo J. H. Kim D. A. Lee J. W. Kim K. I. Lee S. H. (2022). Steep posterior lateral tibial slope, bone contusion on lateral compartments and combined medial collateral ligament injury are associated with the increased risk of lateral meniscal tear. Knee Surg. Sports Traumatol. Arthrosc. 30 (1), 298–308. 10.1007/s00167-021-06504-z 33687540

[B16] Kodama Y. Furumatsu T. Tamura M. Okazaki Y. Hiranaka T. Kamatsuki Y. (2023). Steep posterior slope of the medial tibial plateau and anterior cruciate ligament degeneration contribute to medial meniscus posterior root tears in young patients. Knee Surg. Sports Traumatol. Arthrosc. 31 (1), 279–285. 10.1007/s00167-022-07095-z 35978177

[B17] Kraeutler M. J. Welton K. L. Chahla J. LaPrade R. F. McCarty E. C. (2018). Current concepts of the anterolateral ligament of the knee: anatomy, biomechanics, and reconstruction. Am. J. Sports. Med. 46 (5), 1235–1242. 10.1177/0363546517701920 28426251

[B18] Kujala U. M. Nelimarkka O. Koskinen S. K. (1992). Relationship between the pivot shift and the configuration of the lateral tibial plateau. Arch. Orthop. Trauma. Surg. 111 (4), 228–229. 10.1007/BF00571483 1622714

[B19] Le C. Y. Galarneau J. M. R F. S. Emery C. A. Manns P. J. Whittaker J. L. (2023). Youth with a sport-related knee injury exhibit significant and persistent knee-related quality-of-life deficits at 12-month follow-up compared to uninjured peers. J. Orthop. Sports. Phys. Ther. 53 (8), 480–489. 10.2519/jospt.2023.11611 37339378

[B20] Li K. Zheng X. Li J. Seeley R. A. Marot V. Murgier J. (2021). Increased lateral femoral condyle ratio is associated with greater risk of alc injury in non-contact anterior cruciate ligament injury. Knee Surg. Sports Traumatol. Arthrosc. 29 (9), 3077–3084. 10.1007/s00167-020-06347-0 33170316

[B21] Li L. Yang L. Zhang K. Zhu L. Wang X. Jiang Q. (2020). Three-dimensional finite-element analysis of aggravating medial meniscus tears on knee osteoarthritis. J. Orthop. Transl. 20, 47–55. 10.1016/j.jot.2019.06.007 PMC6939112 31908933

[B22] Li R. Liu Y. Fang Z. Zhang J. (2020). Introducing the lateral femoral condyle index as a risk factor for anterior cruciate ligament injury: letter to the editor. Am. J. Sports. Med. 48 (7), NP42. 10.1177/0363546520920546 32501159

[B23] Li R. Yuan X. Fang Z. Liu Y. Chen X. Zhang J. (2020). A decreased ratio of height of lateral femoral condyle to anteroposterior diameter is a risk factor for anterior cruciate ligament rupture. BMC Musculoskelet. Disord. 21 (1), 402. 10.1186/s12891-020-03440-w 32576249 PMC7313127

[B24] Li W. Liang J. Zeng F. Lin B. Liu C. Huang S. (2021). Anatomic characteristics of the knee influence the risk of suffering an isolated meniscal injury and the risk factors differ between women and men. Knee Surg. Sports Traumatol. Arthrosc. 29 (11), 3751–3762. 10.1007/s00167-020-06396-5 33388828

[B25] Malviya A. Lingard E. A. Weir D. J. Deehan D. J. (2009). Predicting range of movement after knee replacement: the importance of posterior condylar offset and tibial slope. Knee Surg. Sports Traumatol. Arthrosc. 17 (5), 491–498. 10.1007/s00167-008-0712-x 19139846

[B26] Nowak E. K. Beaulieu M. L. Beynnon B. D. Ashton-Miller J. A. Sturnick D. R. Wojtys E. M. (2022). The lateral femoral condyle index is not a risk factor for primary noncontact anterior cruciate ligament injury. Am. J. Sports. Med. 50 (1), 85–92. 10.1177/03635465211057271 34846175 PMC8732325

[B27] Okazaki Y. Furumatsu T. Kodama Y. Kamatsuki Y. Okazaki Y. Hiranaka T. (2021). Steep posterior slope and shallow concave shape of the medial tibial plateau are risk factors for medial meniscus posterior root tears. Knee Surg. Sports Traumatol. Arthrosc. 29 (1), 44–50. 10.1007/s00167-019-05590-4 31243503

[B28] Pfeiffer T. R. Burnham J. M. Hughes J. D. Kanakamedala A. C. Herbst E. Popchak A. (2018). An increased lateral femoral condyle ratio is a risk factor for anterior cruciate ligament injury. J. Bone. Jt. Surg. Am. 100 (10), 857–864. 10.2106/JBJS.17.01011 29762281

[B29] Pfeiffer T. R. Burnham J. M. Kanakamedala A. C. Hughes J. D. Zlotnicki J. Popchak A. (2019). Distal femur morphology affects rotatory knee instability in patients with anterior cruciate ligament ruptures. Knee Surg. Sports Traumatol. Arthrosc. 27 (5), 1514–1519. 10.1007/s00167-018-5269-8 30374573

[B30] Shen L. Jin Z. G. Dong Q. R. Li L. B. (2018). Anatomical risk factors of anterior cruciate ligament injury. Chin. Med. J. Engl. 131 (24), 2960–2967. 10.4103/0366-6999.247207 30539909 PMC6302639

[B31] Snoeker B. A. Bakker E. W. Kegel C. A. Lucas C. (2013). Risk factors for meniscal tears: a systematic review including meta-analysis. J. Orthop. Sports. Phys. Ther. 43 (6), 352–367. 10.2519/jospt.2013.4295 23628788

[B32] Song G. Y. Zhang H. Zhang J. Liu X. Xue Z. Qian Y. (2018). Greater static anterior tibial subluxation of the lateral compartment after an acute anterior cruciate ligament injury is associated with an increased posterior tibial slope. Am. J. Sports. Med. 46 (7), 1617–1623. 10.1177/0363546518760580 29578774

[B33] Vincent J. P. Magnussen R. A. Gezmez F. Uguen A. Jacobi M. Weppe F. (2012). The anterolateral ligament of the human knee: an anatomic and histologic study. Knee Surg. Sports Traumatol. Arthrosc. 20 (1), 147–152. 10.1007/s00167-011-1580-3 21717216

[B34] Voleti P. B. Stephenson J. W. Lotke P. A. Lee G. C. (2014). Plain radiographs underestimate the asymmetry of the posterior condylar offset of the knee compared with mri. Clin. Orthop. Relat. Res. 472 (1), 155–161. 10.1007/s11999-013-2946-2 23536177 PMC3889463

[B35] Wang P. Gao F. Sun W. Li Z. Wu X. Shi L. (2022). Morphometric characteristics of the knee are associated with the injury of the meniscus. J. Orthop. Surg. Res. 17 (1), 498. 10.1186/s13018-022-03380-2 36403063 PMC9675146

[B36] Wong C. K. Man G. He X. Ng J. P. Ng A. Ong M. (2022). Large lateral tibial slope and lateral-to-medial slope difference are risk factors for poorer clinical outcomes after posterolateral meniscus root tear repair in anterior cruciate ligament reconstruction. BMC Musculoskelet. Disord. 23 (1), 247. 10.1186/s12891-022-05174-3 35287650 PMC8922830

[B37] Wu M. Jiang J. Liu Z. Dai X. Dong Y. Xia Y. (2022). Age, male sex, higher posterior tibial slope, deep sulcus sign, bone bruises on the lateral femoral condyle, and concomitant medial meniscal tears are risk factors for lateral meniscal posterior root tears: a systematic review and meta-analysis. Knee Surg. Sports Traumatol. Arthrosc. 30 (12), 4144–4155. 10.1007/s00167-022-06967-8 35429241

[B38] Zhou T. (2018). Analysis of the biomechanical characteristics of the knee joint with a meniscus injury. Healthc. Technol. Lett. 5 (6), 247–249. 10.1049/htl.2018.5048 30568803 PMC6275133

